# Hyperglucagonemia in an animal model of insulin- deficient diabetes: what therapy can improve it?

**DOI:** 10.1186/s40842-016-0029-5

**Published:** 2016-05-02

**Authors:** Fabrizio Barbetti, Carlo Colombo, Leena Haataja, Corentin Cras-Méneur, Sergio Bernardini, Peter Arvan

**Affiliations:** 1 Department of Experimental Medicine and Surgery, University of Tor Vergata, Rome, Italy; 2 Bambino Gesù Children’s Hospital, IRCCS, Rome, Italy; 3 Division of Metabolism, Endocrinology & Diabetes, University of Michigan Medical Center, Ann Arbor, MI USA; 4 Department of Experimental Medicine and Surgery, University of Tor Vergata. Tor Vergata University Hospital, first floor, section D, room 118, Viale Oxford 81, 00133 Rome, Italy; 5 University of Michigan Medical Center, Brehm Tower room 5112, 1000 Wall St., Ann Arbor, MI 48105 USA

**Keywords:** C-peptide I, C-peptide II, Glucagon, Exendin-4

## Abstract

**Background:**

Intra-islet insulin contributes to alpha-cell suppression. *Akita* mice carry a toxic-gain-of- function *Ins2* gene mutation encoding proinsulin-C(A7)Y, similar to that described in human Mutant *Ins*-gene induced Diabetes of Youth, which decreases intra-islet insulin. Herein, we examined *Akita* mice for examination of circulating insulin and circulating glucagon levels. The possibility that loss of intra-islet suppression of alpha-cells, with increased circulating glucagon, contributes to diabetes under conditions of intra-islet insulin deficiency, raises questions about effective treatments that may be available.

**Methods:**

Blood glucose, plasma insulin, C-peptide I, C-peptide II, and glucagon were measured at various times during development of diabetes in *Akita* mice. We also used *Akita*- like hProC(A7)Y-CpepGFP transgenic mice in *Ins2* ^*+/+*^, *Ins2* ^*+/−*^ and *Ins2* ^*−/−*^ genetic backgrounds (providing animals with greater or lesser defects in islet insulin production, respectively) in order to examine the relative abundance of immunostainable intra-islet glucagon-positive and insulin-positive cells. Similar measurements were made in *Akita* mice. Finally, the effects of treatment with insulin, exendin-4, and leptin on blood glucose were then compared in *Akita* mice.

**Results:**

Interestingly, total insulin levels in the circulation were not frankly low in *Akita* mice, although they did not rise appropriately with the onset of hyperglycemia. By contrast, in severely diabetic *Akita* mice at 6 weeks of age, circulating glucagon levels were significantly elevated. Additionally, in *Ins2* ^+/−^ and *Ins2* ^−/−^ mice bearing the *Akita*-like hProC(A7)Y-CpepGFP transgene, development of diabetes correlated with an increase in the relative intra-islet abundance of immunostainable glucagon-positive cells, and a similar observation was made in *Akita* islets. In *Akita* mice, whereas a brief treatment with exendin-4 resulted in no apparent improvement in hyperglycemia, leptin treatment resulted in restoration of normoglycemia. Curiously, leptin treatment also suppressed circulating glucagon levels.

**Conclusions:**

Loss of insulin-mediated intra-islet suppression of glucagon production may be a contributor to hyperglycemia in *Akita* mice, and leptin treatment appears beneficial in such a circumstance. This treatment might also be considered in some human diabetes patients with diminished insulin reserve.

## Background

All forms of diabetes can be said to exhibit insulin deficiency relative to the degree of hyperglycemia. In type 1 diabetes there is absolute insulin deficiency, and in type 2 diabetes there is relative insulin deficiency that may progress to absolute insulin deficiency over time [1, 2]. Intra-islet insulin production is known to have suppressive effects on islet alpha cells [3]. In recent years, through many potential molecular mechanisms, possible contributions of hyperglucagonemia originating from islet alpha cells to the hyperglycemic phenotype of both early stage type 1 diabetes and late stage type 2 diabetes have been suggested [4, 5].

Patients with autosomal dominant Mutant *INS*-gene induced Diabetes of Youth (MIDY [6, 7]) serve as an excellent model of insulin-deficient diabetes. Such mutations affect proinsulin folding and cause insulin-deficient diabetes in both humans and animal models [8–11]. The dominant-negative effect of these mutants is caused by impairment of wild-type proinsulin intracellular transport [12–14] and eventually, the insulin-deficient diabetes is compounded by apoptosis of beta cells that suffer from endoplasmic reticulum (ER) stress [15]. Curiously, in many human patients with permanent neonatal diabetes caused by MIDY mutations, circulating C-peptide is detectable or even high at the time of diabetes onset [11]. On the other hand, little has been reported about circulating glucagon levels in such patients, and currently, treatment options consist mainly of full insulin replacement dosing [11], which of course comes with concomitant risk of unintended – or even fatal – hypoglycemia.

*Akita* mice are an animal model of MIDY, developing early onset diabetes with subsequent diabetes complications. The *Akita* mice are heterozygous for the *Ins2* gene mutation encoding proinsulin-C(A7)Y, and all males bearing the mutation proceed to develop severe diabetes after weaning. The Cys(A7) is also a site of mutation in human MIDY [10]. In *Akita* islets, approximately one third of all newly-synthesized proinsulin is recovered as the mutant form [14] but a second *Akita*-like model bearing the same mutation expressed as a transgene, produces a much lower level of mutant proinsulin expression that more modestly lowers intra-islet insulin and leads only to prediabetes [16]. Animals with intra-islet insulin deficiency are attractive models for pre-clinical studies of adjunctive treatments that might eventually be explored in human MIDY patients, as well as in subsets of type 2 diabetes patients that have diminished insulin reserve [17, 18].

In this study, we have examined circulating insulin, C-peptide, and glucagon levels as a function of age in wild-type and *Akita* mice, and we looked directly at the immunostainable pancreatic alpha:beta cell ratio in mice bearing an *Akita*-like mutant proinsulin transgene in place of one or two endogenous *Ins2* alleles [16] — or in the islets of authentic *Akita* mice. Both mouse models suggest loss of insulin-mediated intra-islet suppression of glucagon production, and with this in mind, we have made a preliminary exploration of leptin treatment in *Akita* mice, intended to suppress the hyperglycemia with hyperglucagonemia.

## Methods

### Mice

*Akita* mice (strain name = C57BL/6 J-*Ins2Akita*) were purchased from Jackson Lab and the colony maintained by breeding heterozygous males with wild type females. Progeny were screened according to the Jackson Lab protocol. hProC(A7)Y-CpepGFP mice in *Ins2* ^*+/+*^, *Ins2* ^*+/−*^ and *Ins2* ^*−/−*^ genetic backgrounds have been characterized previously [16, 19]. All experiments were performed on male mice because they develop diabetes in the *Akita* strain background. For C-peptide I and II analyses, serum was collected from heart of unstarved mice; before cardiac blood withdrawal, mice were anesthetized with Avertin. Mice were euthanized by cervical dislocation**.**

### Treatments

Exendin-4 (Sigma #E7144-1 mg) was delivered by injection of 10 nmol/kg body weight once daily for 13 days. The length of this treatment was based on previous work showing that effects of exendin-4 on diabetic mice are manifest within a week [20]. For leptin administration, an Alzet mini-osmotic pump (model 1002) was implanted subcutaneously in 18–20 day-old mice, delivering saline solution (sham) or ~6 mg/day of mouse recombinant leptin (R&D Systems #498-OB) for 2 weeks. For insulin treatment, a LinBit insulin half-dose pellet (LinShin, Canada) was implanted subcutaneously in 18–19 day-old mice that provided an insulin delivery rate of 0.05 U/day for at least 20 days. A second equivalent half-dose pellet was then implanted at 25–26 days and a full-dose pellet implanted at 32–33 days. Negative controls were sham-operated mice (anesthetized, cut, and sutured).

### Biochemical assays

Blood sampling was performed by tail vein bleed from unanesthetized, unstarved mice and analyzed immediately for glucose concentration by the glucose oxidase method using a One Touch II (Lifescan) glucometer. For C-peptide I and II analyses, in the case of young animals, sera were pooled from several mice in order to reach the 200 μL required for immunoassay. For fasting glucagon and insulin analyses, sera were collected from mice starved for 5 h. ELISA kits for mouse C-peptide I, C-peptide II, pancreatic glucagon, and total insulin were purchased from Alpco Diagnostics (respectively #48-CP1MS-E01, #48-CP2MS-E01, #48-GLUHU-E01, #80-INSMS-E01) with the assays performed according to the manufacturer’s instructions.

### Oral glucose tolerance tests (OGTTs)

OGTTs were performed 9 days after surgery. After a 5 h fasting period, a glucose solution at 2 g/kg body weight was administered via gavage, with blood glucose concentrations determined at the indicated time points.

### Immunofluorescence analyses

Following euthanasia, the pancreas of each mouse was rapidly immersion-fixed in formaldehyde, embedded in paraffin and cut in sections, rehydrated in a graded series of alcohols, washed in H_2_O followed by antigen retrieval (Retrieve-ALL.1, Covance) and immunostained with mouse anti-glucagon (Abcam, 1:500) and guinea pig anti- insulin (Linco, 1:1000). The appropriate secondary antibodies were conjugated to AlexaFluor- 555 or 647 (Invitrogen). Slides were mounted with Prolong Gold (Invitrogen) and imaged by epifluorescence in an Olympus FV500 confocal microscope with 60x (NA 1.4) oil objectives. The number of alpha cells (defined as glucagon-positive) or beta cells (defined as insulin- positive) were either manually counted for each islet by a single observer in a blinded fashion and the fraction of each cell type subjected to a Kruskal-Wallis test with Dunn’s corrections for multiple testing (GraphPad Prism 6.0), or was counted by ImageJ-1.50 with the assistance of either the “cell counter plug-in” (http://rsbweb.nih.gov/ij/plugins/cell-counter.html) or with the “ITCN 1.6 plug-in” (http://rsb.info.nih.gov/ij/plugins/itcn.html) and the ratio between the cell types analyzed by Mann-Whitney test (GraphPad Prism 6.0). Cell ratios within individual islets were quantified and expressed as mean ± SE, with *p*-value < 0.05 considered statistically significant.

## Results

### Onset of diabetes in *Akita* mice

**Fig. 1:**
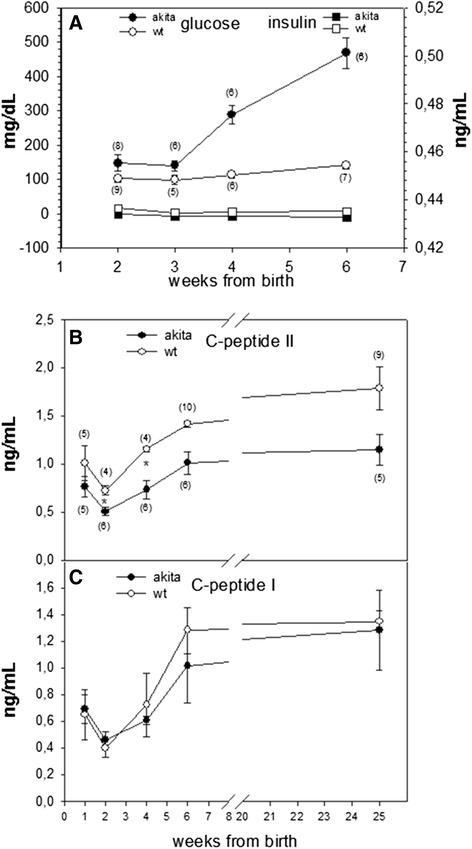
Blood glucose and circulating total insulin, C-peptide II and C-peptide I levels in Akita and wild-type mice. **a** Blood glucose is higher in *Akita* mice from 4 weeks of age (*: *p* < 0.05), while circulating insulin was not frankly low. **b** C-peptide II was consistently lower in *Akita* mice (*: *p* < 0.05 at 2 and 4 weeks of age; *p* = 0.07 at 25 weeks of age). **c** C-peptide I does not show a compensatory increase in *Akita* mice. The number of mice utilized at each point are indicated in parenthesis

The expression of proinsulin-C(A7)Y in *Akita* mice impairs proper folding of the mutant proinsulin, functioning as a dominant-negative that entraps proinsulin within the endoplasmic reticulum of pancreatic beta cells and thereby decreases insulin production [21, 22]. To evaluate the onset of diabetes in *Akita* animals, we followed changes in blood glucose in a cohort of mice while simultaneously measuring serum insulin levels. At a time just before blood glucose began to rise at the 3rd week of age (Fig. 1a, closed circles) insulin levels were not frankly low (Fig. 1a, closed squares).

To get an independent estimate of insulin secretion during onset of diabetes, we also followed circulating levels of the secreted C-peptides of the two non-allelic *Ins* genes; specifically, we measured C-peptide I and C-peptide II for up to 25 weeks of age. The *Akita* mutant proinsulin is the product of one allelic copy of the *Ins2* gene, thus it is not surprising that C-peptide II levels in *Akita* males were significantly lower that that of wild-type animals even at two weeks of age — and remained low for the entire observation period (Fig. 1b). Additionally, circulating C- peptide I did not compensate for the loss of C-peptide II (Fig. 1c), suggesting that insulin I secretion was limited from reaching the expected maximum in these animals that do not secrete adequate levels of insulin II [23]. Moreover, in 2–4 week-old *Akita* mice, neither circulating insulin nor circulating C-peptide rose above wild-type levels despite the development of hyperglycemia that should stimulate increased insulin secretion. Nevertheless, in *Akita* animals, the initial onset of hyperglycemia could not be traced to an actual drop in circulating insulin levels (Fig. 1a), and upon development of hyperglycemia, both C-peptides I and II were in fact able to respond with an increased (albeit inadequate) output up to 6 week of life and beyond. Taken together, the data in Fig. 1 confirm that during onset of diabetes, the islets of *Akita* mice have relative insulin deficiency with diminished insulin reserve.

### Loss of insulin-mediated intra-islet suppression of glucagon production in *Akita* mice

**Fig. 2:**
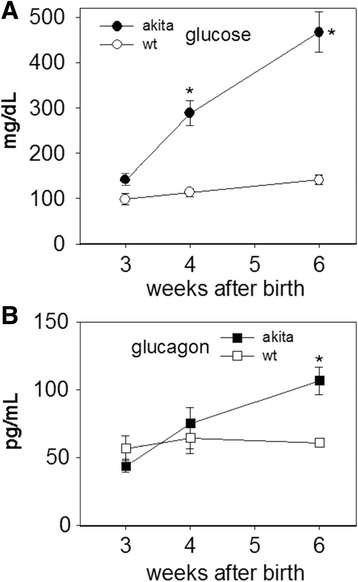
Blood glucose (panel **a**) and circulating glucagon levels (panel **b**) in *Akita* mice and wild type mice from 3 to 6 weeks of age. Glucagon rises along with an increase in blood glucose increase in *Akita* mice (5 mice): *: *p* < 0.005 for both glucose and glucagon at 6 weeks of age when compared to wild type animals (8 mice)

We previously reported that patients with permanent neonatal diabetes mellitus (PNDM) due to proteotoxic MIDY mutations have fasting hyperglucagonemia [11], suggesting loss of insulin- mediated intra-islet suppression of glucagon production [24, 25]. In turn, increased glucagon levels may drive an increased rate of gluconeogenesis and glycogenolysis, contributing to hyperglycemia [24–26]. *Akita* mice have a normal blood glucose for up to three weeks of age (Fig. 1a); we therefore examined circulating glucagon levels weekly beginning at this time. Interestingly, we found that circulating glucagon levels showed an increase between 3 and 4 weeks that rose along with the initial rise of blood glucose into the diabetic range (Fig. 2). Indeed at 6 weeks of age when *Akita* animals had random blood glucose levels ≥ 450 mg/dL, plasma glucagon was significantly elevated (Fig. 2b).

**Fig. 3:**
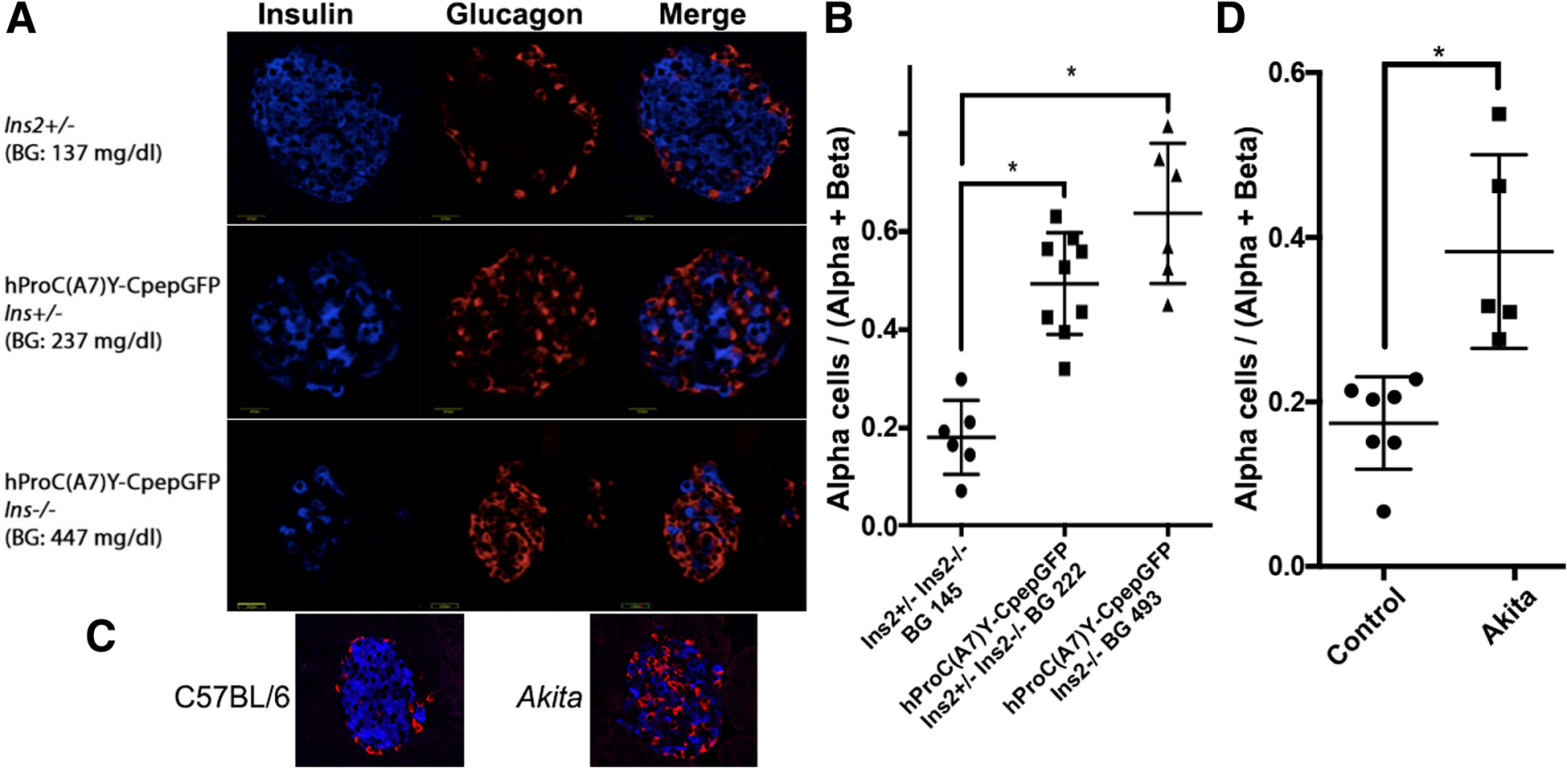
Increase in the relative abundance of immunostainable alpha cells in hProC(A7)Y- CpepGFP mice with 1 or 2 functional *Ins2* alleles deleted, or in authentic *Akita* mice. **a** Paraffin sections of pancreata from mice with the genotypes indicated, were immunostained with anti-insulin (blue) and anti-glucagon (red). Scale bar: 20 μm. **b** From confocal microscope images, a blinded reader scored the number of glucagon-positive and insulin- positive cells. With ~1000 immunostainable cells per mouse, the fraction of immunostainable cells that were glucagon-positive (called “Alpha”) was quantitated for each mouse (shown as individual points in scatter plot with mean ± SE; *: *p* < 0.05. As noted in the Results, we did not observe double hormone-positive cells. **c** Paraffin sections of pancreata from C57Bl/6 control or *Akita* diabetic (male) mice were immunostained as in panel A, with representative images shown. **d** From control and *Akita* diabetic mice, the fraction of immunostainable cells that were glucagon-positive (called “Alpha”) was quantitated and presented as a scatter plot as in panel B, with mean ± SE; *: *p* < 0.05

As noted in the Background section, whereas *Akita* islets synthesize approximately one third of their proinsulin as the mutant protein [14], transgenic hProC(A7)Y-CpepGFP mice express lower levels of *Akita*-like mutant proinsulin that only modestly lowers intra-islet insulin levels and results only in prediabetes — expression of the transgene in *Ins2* ^*+/−*^ and *Ins2* ^*−/−*^ genetic backgrounds [23] increases the severity of the diabetes phenotype [16]. We examined pancreata from such mice stratified by their random blood glucose levels. With beta cells that were easily recognized by their GFP fluorescence, we immunostained random pancreas sections containing islet cross-sectional images to identify glucagon-expressing cells (considered to be alpha cells) — counting approximately 1000 immunostainable cells per each mouse. Of the *Ins2* ^+/−^ or *Ins2* ^−/−^ mice in the absence of the *Akita*-like transgene, all were normoglycemic (random BG 145 ± 27 mg/dl) and ~20 % of the sum of beta + alpha cells in these islets were alpha cells (Fig. 3a upper row, b). Mice bearing the *Akita*-like hProC(A7)Y-CpepGFP transgene in an Ins2^+/−^ background mostly exhibited mild hyperglycemia (random BG 222 ± 18 mg/dl) and from the sum of beta + alpha cells in these islets, ~ half were alpha cells (Fig. 3a middle row, b). Of those mice bearing the *Akita*-like hProC(A7)Y-CpepGFP transgene in the *Ins2* ^−/−^ background (and occasionally from the *Ins2* ^+/−^ background), animals with frank diabetes emerged (random BG 493 ± 57 mg/dl). In these animals, we measured ~60 % alpha cells; Fig. 3a bottom row, b).

We then repeated the same immunostaining analysis using pancreas sections from authentic *Akita* diabetic (male) mice compared to C57BL/6 (male) littermate controls. As has been reported many times in control mouse islets, the glucagon-expressing cells were found at the perimeter of the islet with beta cells concentrated in the central core — we observed this same feature in both in the C57BL/6 control animals and in our *Ins2* ^+/−^ control mice (Fig. 3a, c).

However, in authentic *Akita* diabetic mice, as in our *Akita*-like transgenic diabetic mice, in addition to the alpha cells at the islet perimeter was a consistent increase in the number of additional glucagon-expressing cells in the islet interior (Fig. 3a, c). Authentic *Akita* diabetic mice exhibited severe hyperglycemia (average random blood glucose 532 mg/dL) and from the sum of beta + alpha cells in these islets, once again, the relative alpha cell abundance more than doubled (Fig. 3d). We did not observe double hormone-positive cells. Together, the data in Figs. 2 and 3 are consistent with the notion that in the presence of misfolded proinsulin that decreases intra-islet insulin, there is loss of insulin-mediated intra-islet suppression of glucagon production, which correlates with onset of diabetes.

### Diabetes treatment in animals with diminished insulin reserve and hyperglucagonemia

**Fig. 4:**
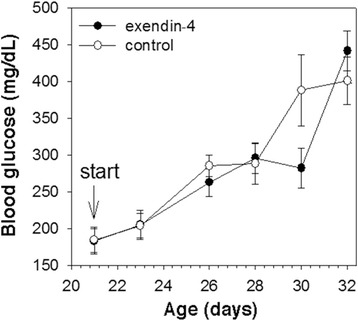
Treatment of *Akita* mice with exendin-4 (4 mice) or sham-treated (physiologic solution; 7 mice) beginning at 3 weeks of age, as noted with arrow

On the basis of the foregoing findings, we proceeded to examine the efficacy of various potential therapies for diabetes in *Akita* mice. First, we tested the effects of exendin-4, a GLP-1 analogue that is thought to help stimulate insulin secretion while suppressing glucagon secretion [27–30]. Following a drug administration protocol similar to that used to achieve glucose lowering in nonobese diabetic mice [30], *Akita* mice were treated with exendin-4 (10 nmol/kg body weight) or vehicle once a day for 13 days starting from the 3rd week of age. Unfortunately, exendin-4 treatment failed to control the development of hyperglycemia in these animals (Fig. 4).

**Fig. 5:**
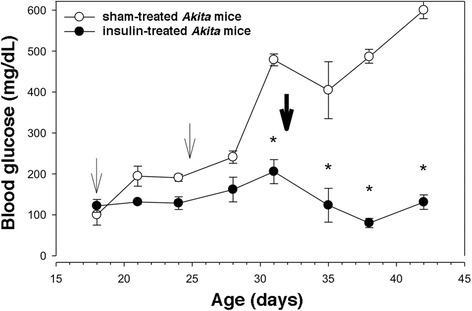
Treatment of *Akita* mice between 18 and 42 days of life with long-acting insulin pellets at half-dose (6 animals, *light arrows*) and full-dose (bold arrow). *: *p* < 0.005 compared to sham-treated animals (8 mice)

As a positive control, we looked at effects of long-acting insulin administration to *Akita* mice. However, without frequent dose adjustment — similar to humans with permanent neonatal diabetes and type 1 diabetes — smooth glycemic control was difficult to achieve. The insulin half-dose pellets we used provide an insulin infusion rate comparable to ~0.05 U/24 h. We administered half-dose insulin pellets at days 18 and 25. Sham-treated *Akita* mice developed full-blown diabetes when random glucose was measured on day 31, whereas long-acting half- dose insulin clearly prevented onset of hyperglycemia **(**Fig. 5). However, when full-dose insulin pellets were administered beginning at day 32 day in an attempt to maintain relatively constant insulin dosing per gram of body weight as the animals grew, hypoglycemia ensued (Fig. 5), which was fatal for several animals. These results highlight the relatively narrow therapeutic window for long-acting insulin treatment.

**Fig. 6:**
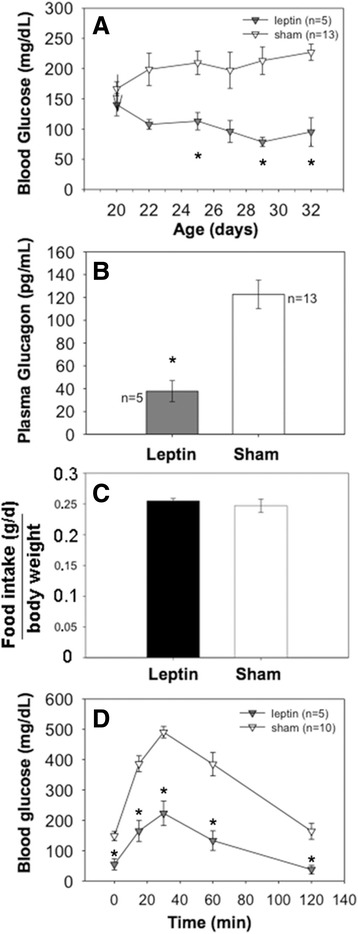
Effect of leptin treatment in *Akita* mice. **a** Five mice had implanted the osmotic pump containing leptin; 13 mice were sham-operated. At 9 days after initiating treatment, blood glucose was significantly lower in leptin-treated animals, *: *p* < 0.05; at 22 days of age *p* = 0.06; at 27 days of age *p* = 0.07. **b** Glucagon secretion is blunted during leptin treatment, *p: < 0.0009. Plasma glucagon was measured 12 days after the osmotic pump containing leptin was implanted. **c** No differences in food intake were found between leptin-treated (5) or sham- operated (13) *Akita* mice. **d** OGTT in leptin-treated (5) and sham-operated (10) *Akita* mice. *: *p* < 0.05

Finally, the effects of leptin [31] were assessed. *Akita* males (mean age: 20 d) were implanted with an osmotic pump delivering recombinant mouse leptin at ~0.7 mg per g body weight per day (a dose comparable to that used in adult NOD or streptozotocin-treated mice [32, 33]). This treatment elicited a significant and sustained decrease in blood glucose (Fig. 6a). Interestingly, preservation of normoglycemia in leptin-treated *Akita* mice was associated with a significant decrease of circulating glucagon (Fig. 6b) but could not be explained by decreased food intake (Fig. 6c). Oral glucose tolerance tests performed 9 d after initiating leptin treatment showed glycemic improvement at all time points (Fig. 6d). Most significantly, all of the leptin-treated animals appeared active and healthy. Taken together, the data in Fig. 6 support the notion that leptin treatment may be of therapeutic value in subjects with diminished insulin reserve, and that suppression of alpha cell glucagon production may be one potential mechanism by which leptin could be effective in controlling hyperglycemia.

## Discussion

Patients with MIDY become progressively insulinopenic (and may even present with diabetic ketoacidosis – signifying insulin deficiency) [11]. *Akita* mice, and “Munich” mice [9] – two animal models of MIDY – also develop insulin deficiency [34, 35]. This was formally established in our own *Akita* colonies not only by circulating insulin levels that did not rise with onset of hyperglycemia, but also by circulating C-peptide I and II (the same parameters followed in human patients) that may more closely reflect insulin secretion rates. Of course, the product of the mutant *Ins2* allele in *Akita* mice cannot be secreted [8] and this alone can lower the circulating level of C-peptide II (Fig. 1b). Additionally, despite a compensatory increase of transcription and translation of the *Ins1* gene in animals with decreased production of the *Ins2* gene product [23], circulating C-peptide I levels did not demonstrate any compensatory increase (Fig. 1c). Proteotoxicity of misfolded proinsulin in *Akita* mice may be less severe than in some humans with MIDY because of the lower mutant gene dosage in mice (that have three additional wild-type *Ins* gene alleles as compared to humans that have only one additional wild-type allele); thus, the *Akita* diabetic mouse reflects a model of individuals with misfolded proinsulin and diminished intra-islet insulin reserve. Indeed, we also examined transgenic hProC(A7)Y- CpepGFP mice in *Ins2* ^*−/−*^ and *Ins2* ^*+/−*^ genetic backgrounds, to further lower the relative expression of misfolded proinsulin, which is a means to limit the inhibition of intra-islet insulin production [16, 19].

The combined action of insulin and glucagon provide coordinate control of hepatic glucose production, such that even small changes in the circulating glucagon/insulin ratio may affect glycemic control. Hyperglucagonemia, which is believed to derive mainly from a loss of insulin-mediated intra-islet suppression of glucagon production [24, 25, 36], is increasingly recognized as an important cause of hyperglycemia [26], and we have reported hyperglucagonemia as a phenotypic feature in MIDY patients [11].

Herein, we report that circulating glucagon in *Akita* mice [35] rises with the onset of diabetes (Fig. 2). Correlating positively with the development of hyperglycemia is an increase in the relative abundance of immunostainable glucagon-positive cells within the islets of mice bearing misfolded mutant proinsulin (Fig. 3). There are a number of important caveats, however. First, as we used random pancreatic sections (counting approximately 1000 immunostainable cells for each mouse) rather than a systematic analysis of islets from the head, body, and tail in which it could be possible that there may be differences in relative abundance of glucagon-positive and insulin-positive cells. Additionally, as the *Akita* or *Akita*-like transgenic mice progress to diabetes, the relative abundance of glucagon-positive cells is likely to change, and thus the ratios that we have measured are not absolute. However, we do note from our scatter plots (Fig. 3c, d) that the relative abundance of alpha cells is quite similar from each independent mouse within each group.

Importantly, in both wild-type C57BL/6 control animals and in our *Ins2* ^+/−^ control mice, alpha cells were found at the perimeter of the islets with beta cells concentrated in the central core. By contrast, in authentic *Akita* diabetic mice and in our *Akita*-like transgenic diabetic mice, in addition to the alpha cells at the islet perimeter was the increased number of glucagon- expressing cells in the islet interior which can account for why the relative alpha cell abundance more than doubled in the animals with intra-islet insulin deficiency (Fig. 3). Our results appear strikingly similar to a recent study of experimental insulin deficiency generated by inducible deletion of insulin genes, in which islet cells deficient for insulin expression were found to mis-express glucagon — and those glucagon-positive cells were located in the islet core — causing the glucagon-positive area (as well as glucagon mRNA) to more than double [37]. Thus, an unanswered question for future research is whether actual expansion of the alpha cell population occurs, or whether there is reprogramming of beta cells (or other cells) to make more glucagon. Indeed, additional studies are still needed to explore the precise relationships between loss of beta cell insulin production, glucagon-positive cell mass, hyperglucagonemia, and the onset of hyperglycemia in insulin-deficient diabetes.

Insulin is currently the only therapy employed for MIDY patients. Insulin therapy may relieve the burden of proteotoxic insulin in the endoplasmic reticulum [38], but this of course comes with a well known risk of hypoglycemia that can be fatal. In this study, we have made a preliminary exploration of treatments intended to suppress hyperglucagonemia and control diabetes in conditions of diminished insulin reserve [39–42] without the risk of fatal hypoglycemia. Specifically in *Akita* mice, we compared the effects of the subcutaneous administration of exendin-4 [27–30] to those of leptin. Unfortunately, exendin-4 was not efficacious for the treatment of diabetes in *Akita* mice (Fig. 4). This may be explained by the fact that only 20 % of glucagon-positive rodent islet cells colocalize with GLP-I receptor immunoreactivity from which a direct action of GLP-1 may occur [43]. Notably a recent study looked directly at acute effects of subcutanous exendin-4 treatment in wild-type mice (as well as insulin-deficient Wolfram syndrom mice) and found no decrement of circulating glucagon levels [44]. Interestingly, in mice, exendin-4 does not increase immunostainable insulin-positive islet area, or decrease immunostainable glucagon-positive islet area. [45] Whether other treatment protocols or other glucagon-like peptide 1 receptor agonists might yield a different outcome remains to be determined, whereas effective suppression of islet glucagon production is an established consequence of enhanced intra-islet insulin production [46]. Based on our present findings we hypothesize that — at least in mice — exendin-4 actions on beta cells may not upregulate insulin production when that insulin production is intrinsically limited by the presence of significant quantities of misfolded proinsulin.

By contrast, we were encouraged by the results of leptin treatment [47] which clearly prevented onset of diabetes in *Akita* mice without inducing fatal hypoglycemia (Fig. 6a). In parallel with the suppression of diabetes was the suppression of circulating glucagon levels (Fig. 6b). Thus, our results highlight that inhibition of alpha cell glucagon production is one potential mechanism by which leptin might be effective in controlling hyperglycemia in individuals with diabetes caused by diminished insulin reserve. With this in mind, we note that leptin receptors are present on primary mouse alpha cells and that leptin can directly suppress electrical activity of these cells to inhibit glucagon secretion [48]. Additionally, incubation of isolated mouse islets with leptin for 24 h dramatically decreases glucagon mRNA (similarly to the effect of insulin) and also diminishes glucagon protein level in the islets [49]. Moreover, lowering of pancreatic and circulating glucagon was also observed in *Akita* mice bioengineered for hepatic secretion of leptin via a transgene that raised circulating leptin levels to those seen in obese animals [47]. Similar to our results, these authors reported that *Akita* mice have increased immunostainable glucagon-positive cell area per islet, and they found that transgenic leptin expression in the *Akita* mice resulted in a significant decrease in immunostainable glucagon- positive cell area per islet (with a nonsignificant increase in immunostainable beta cell area per islet). Taken together, the current evidence suggests that suppression of exuberant islet glucagon production in *Akita* mice can be improved by leptin treatment.

Published results of others suggest that this approach may have promise for humans [31] as well as rodents [50, 51]. While no undesirable actions of leptin have yet been reported in decade- long replacement therapy in patients with mutation of the leptin gene [52], and Metreleptin is now approved by the U.S. Food and Drug Administration (FDA) for the treatment of severe metabolic abnormalities associated with lipodystrophy [53], its efficacy (and safety) in other metabolic disorders will still need to be studied. Specifically, studies examining the possible utility of combined insulin and leptin treatment for control of both hyperglycemia and hyperglucagonemia in insulin-deficient diabetes may be of particular value. In addition, other pharmacologic agents with specific alpha cell-suppressive actions also deserve further pursuit.

## Conclusions

Loss of insulin-mediated intra-islet suppression of glucagon production appears to be a contributor to the hyperglycemia of *Akita* mice, a model of insulin-deficient diabetes. Leptin treatment appears to be beneficial in such a circumstance, thus this treatment might also be considered in some human diabetes patients with diminished insulin reserve.

### Approval of animal procedures

Principles of laboratory animal care (NIH publication no. 85–23, revised 1985) were followed at all times, and all animal protocols were approved by the relevant institutional animal care and use committee.

## Abbreviations

MIDY: mutant ins-gene induced diabetes of youth
